# *Garcinia cambogia* Phenolics as Potent Anti-COVID-19 Agents: Phytochemical Profiling, Biological Activities, and Molecular Docking

**DOI:** 10.3390/plants11192521

**Published:** 2022-09-26

**Authors:** Hanan Y. Aati, Ahmed Ismail, Mostafa E. Rateb, Asmaa M. AboulMagd, Hossam M. Hassan, Mona H. Hetta

**Affiliations:** 1 Department of Pharmacognosy, College of Pharmacy, King Saud University, P.O. Box 2457, Riyadh 11451, Saudi Arabia; 2 Pharmacognosy Department, Faculty of Pharmacy, Fayoum University, Fayoum 63514, Egypt; 3 School of Computing, Engineering & Physical Sciences, University of the West of Scotland, Paisley PA1 2BE, Scotland, UK; 4 Department of Pharmaceutical Chemistry, Faculty of Pharmacy, Nahda University (NUB), Beni-Suef 62513, Egypt; 5 Pharmacognosy Department, Faculty of Pharmacy, Beni-Suef University, Beni-Suef 62513, Egypt

**Keywords:** *Garcinia cambogia*, anti-COVID-19, 3CL-pro, molecular docking, chemical profiling

## Abstract

COVID-19 is a disease caused by the coronavirus SARS-CoV-2 and became a pandemic in a critically short time. Phenolic secondary metabolites attracted much attention from the pharmaceutical industries for their easily accessible natural sources and proven antiviral activity. In our mission, a metabolomics study of the *Garcinia cambogia* Roxb. fruit rind was performed using LC-HRESIMS to investigate its chemical profile, especially the polar aspects, followed by a detailed phytochemical analysis, which led to the isolation of eight known compounds. Using spectrometric techniques, the isolated compounds were identified as quercetin, amentoflavone, vitexin, rutin, naringin, catechin, *p*-coumaric, and gallic acids. The antiviral activities of the isolated compounds were investigated using two assays; the 3CL-M^pro^ enzyme showed that naringin had a potent effect with IC_50_ 16.62 μg/mL, followed by catechin and gallic acid (IC_50_ 26.2, 30.35 μg/mL, respectively), while the direct antiviral inhibition effect of naringin confirmed the potency with an EC_50_ of 0.0169 μM. To show the molecular interaction, in situ molecular docking was carried out using a COVID-19 protease enzyme. Both biological effects and docking studies showed the hydrophobic interactions with Gln 189 or Glu 166, per the predicated binding pose of the isolated naringin.

## 1. Introduction

COVID-19 is a novel severe acute respiratory syndrome coronavirus 2 (SARS-CoV-2). It is a contagious strain of coronavirus that appeared in Wuhan, China in December 2019. The virus is believed to be of animal origin, emerging from the animal market through human−animal contact and causing respiratory and gastrointestinal tract infections with various symptoms starting from fever and general fatigue to shortness of breath, respiratory issues, renal failures, and death [1].

The SARS-CoV-2 virus infection spreads by person-to-person contact with another contaminated person. Serious complications could happen to immune-compromised individuals and the older population, who can develop a critical condition [2]. According to WORLDMETER records (12 September 2022), more than 614 million cases have been reported since the start of this pandemic [3]. WHO also recorded over 5.7 million new cases in the first week of July 2022, and over 6.3 million deaths have been reported globally [4]. Billions of COVID-19 vaccines were aligned with drug protocols to fight this global pandemic.

Coronaviruses cause multiple respiratory and intestinal infections in both humans and animals. The 3-chymotrypsin-like cysteine protease (3CL^Pro^), known as main protease (M^pro^), plays an essential role in the development of the polyproteins that result from the viral RNA translation [5]. Consequently, 3CL^Pro^ inhibitors are a promising choice that can block viral replication and be used to treat COVID-19 patients. The 3CL protease assay used has the advantage in that it is a homogeneous assay with no time-consuming washing steps.

Nowadays, the importance of phytomedicine against COVID-19 has gained much attention by using either plants’ crude extracts or their pure isolates. Many publications revealed the role of some plants against different respiratory viruses, including COVID-19, through many mechanisms, such as blocking virus release or even inhibiting enzymes responsible for replication and viral entry inhibition. Examples of these plants and their active metabolites include *Pelargonium sidoides, Isatis indigotica, Artemisia annua*, and glycyrrhizin from *Glycyrrhiza glabra* roots [6].

The Guttiferae (Clusiaceae) is a tropical family comprising about 37 genera and 1610 species of trees and shrubs [7]. The genus *Garcinia* contains a large number of bioactive secondary metabolites. It has received considerable attention for its chemical content in the last few years as its extracts are rich in derivatives of polyphenols, polyisoprenylated benzophenones, xanthones, and bioflavonoids [8]. Phenolic compounds have important functional properties, such as antioxidant, anticancer, and anti-inflammatory capacities, among others [9]. In a recent publication, the garcinoic acid in *Garcinia kola* suppressed the induced hyper-inflammatory SARS-CoV-2 spike glycoprotein S1 by suppressing the activation of NF-κBin human PBMCs [10].

*Garcinia cambogia* Roxb. (synonymous to *Garcinia cowa* Roxb.) is native to southeast Asia and commonly known as Malabar tamarind. It was reported in a recent publication [8] that its fruits are rich in hydroxycitric acid, which has an antiobesity effect, as well as oxy-guttiferone-K, M, and I with their benzophenones’ analogues, which are topoisomerase II inhibitors. Benzophenones garcinol, rheediaxanthone-A, and isogarcinol were also identified from its peel and volkensiflavone and fukugetin bioflavonoids from its pericarp.

Previous investigations of the antioxidant, antileukemic, antifungal, antiviral, antibacterial, hypolipidemic, and antiadipogenic activities of the *Garcinia cambogia* Roxb. plant extracts have been conducted [8]. Biological studies on seed extracts showed that they have vasodilatory, antiulcerogenic, and antihistaminic effects. The gastroprotective effects were assumed to diminish acidity and improve the mucosal defenses [11]. A photograph of the fruit is provided in Figure S1.

The objective of this work was to assess the potential of *Garcinia cambogia* Roxb. as a source of phenolic compounds, including its chemical characterization and evaluation of antiviral activities against COVID-19.

## 2. Results

### 2.1. Metabolomic Analysis

Chemical profiling of the secondary metabolites of the aerial parts of crude ethanolic extract of the *Garcinia cambogia* Roxb. fruit rind by the use of LC-HRESIMS led to the identification and characterization of 35 various metabolites (1–35) belonging to various chemical classes, among which phenolics, such as flavonoids, catechins, and phenolic acids, dominated. The identification of the resulted metabolites was processed by applying algorithms and macros, which coupled MZmine with both in-house and online databases (METLIN and DNP databases for plant natural products) (Table S1, Figure S2 in the supplementary file).

### 2.2. Phytochemical Analysis

**Figure 1:**
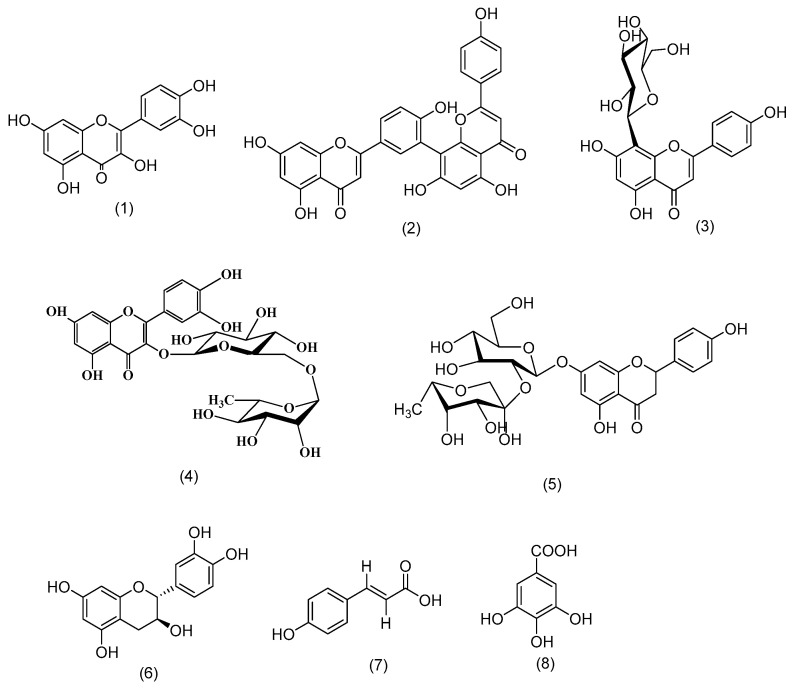
Structures of isolated compounds from the fruit of *Garcinia cambogia* Roxb.: quercetin (**1**), amentoflavone (**2**), vitexin (**3**), rutin (**4**), naringin (**5**), catechin (**6**), *p*-coumaric acid (**7**), gallic acid (**8**).

Eight compounds from different classes were isolated and purified using various chromatographic techniques. The isolated compounds were characterized by their m.p., chromatographic behavior, and different spectral data. Structure elucidation and identification of the compounds were carried out by interpreting their spectral data, comparing them with the literature, and confirming them by the previous metabolomic analysis that indicated all compounds were from the positive ion mode except compound **7**, which was identified from the negative mode. These compounds (**1**–**8**) were identified as quercetin [12], amentoflavone [13], vitexin [13], rutin [12], naringin [13], catechin [13], *p*-coumaric acid [13], and gallic acid [12] (Figure 1).

### 2.3. Biological Activities

#### 2.3.1. 3CL Protease SARS-CoV-2 Activity (M^Pro^ Assay)

The inhibition of SARS-CoV-2 3CL^Pro^; IC_50_ activity by the eight isolated compounds was tested at a final concentration of 50 μM. It was noticed, however, that the incubation of the 3CL^Pro^ with the compound **5** produced a significant reduction of the protease activity compared to the plumbagin positive standard followed by both compounds **6** and **8**, while other compounds showed moderate activities (Table 1, Figure S3). Detailed results and standard curves can be found in the supplementary file (Table S2, Figure S4).

#### 2.3.2. Human Coronavirus (COVID-19) Antiviral Assay

**Figure 2:**
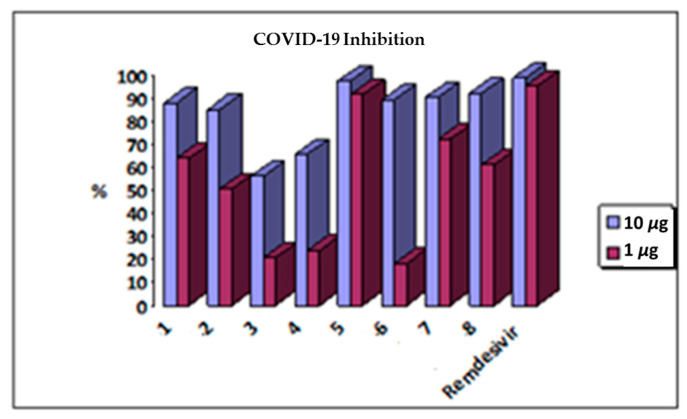
Effect of *Garcinia cambogia* Roxb. isolated compounds on COVID-19.

An antiviral evaluation of the isolated compounds against the coronavirus was performed with two concentrations (1 mM and 10 μM); it was noted that compound **5** possessed a potent inhibition activity of COVID-19 in both test concentrations compared to the remdesivir positive standard. This potency forced us to repeat this test on it to evaluate its EC_50_ with a serial concentration (10, 1, 0.1, 0.01, and 0.001 μM), which confirmed its anti-COVID efficacy (Table 2, Figure 2 and Figure S5). Detailed results and standard curves can be found in the supplementary file (Tables S3 and S4, Figure S6).

### 2.4. Molecular Docking Studies

Docking scores for the natural isolated compounds were provided with the ligand to draw a predictive picture of where these target compounds are binding with the COVID-19 protease (Table 3).

**Figure 3:**
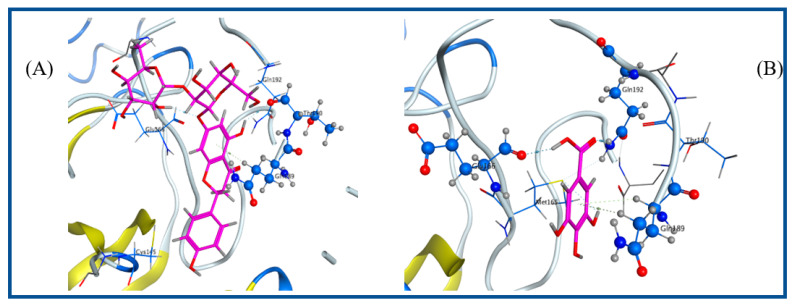
Crystal structure of the predicted binding pose (in blue) of Compound **5** (**A**) and Compound **8** (**B**) with the COVID-19 protease (PDB code: 5R7Z).

**Figure 4:**
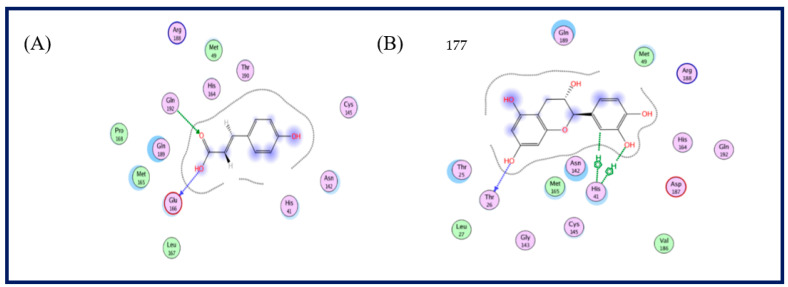
2D structure of the predicted binding pose of compound **7** (**A**) and compound **6** (**B**) with the COVID-19 protease (PDB code: 5R7Z) amino acid residues.

A literature survey of the SARS-CoV-2 main proteases revealed that a relative hydrophobic group is essential for the activity to bind to Gln 189 residue, and the presence of Gln 191 and Glu 166 can form the hydrophobic subsite in other SARS-CoV-2 main proteases, which may create extra van der Waal interactions in addition to one hydrogen bond interaction with Thr 190 [14]. In this study, most of the target compounds showed hydrophobic interactions with Gln 189 or Glu 166. As per the predicted binding pose of compound **5**, one hydrogen bond donor between the hydroxyl phenolic group and the Thr 190 amino acid residue was recognized in addition to a hydrophobic interaction with the Gln 189 residue. Moreover, compound **8** formed the traditional hydrophobic interaction with Gln 189 in addition to two hydrogen bond interactions with Gln 192 and Glu 166. The extra binding interaction of compound **8** may explain the partial increase of vial inhibition. Compound **7** showed two hydrogen bond interactions via binding hydroxyl and carbonyl groups with Glu 166 and Gln 192 residues, respectively. Finally, compound **6** bound to Thr 26 and His 41 via hydrogen bond and hydrophobic interactions (Figure 3 and Figure 4).

## 3. Discussion

Several reports of phenolic natural compounds with anti-SARS-CoV-2 activity indicated metabolites that have been or are currently being studied. Flavonoids are considered natural biologically active phenolic compounds, their antioxidant power due to the presence of hydroxyl functional groups, which enable them to be used as anticancer and antiviral agents by preventing the infection and enhancing immunity. They affect coronaviruses at all stages during their life cycle, starting with cell penetration and the entry inside it, replication of the coronavirus’s nucleic acid, and finally, release of the virion outside the cell; they also could have a role with cellular targets of the host [15]. These phenolics could be an essential source in the battle against coronaviruses with advantageous safe administration without systemic toxicity. This work highlights the significance of phenolics, including flavonoids and their potential on various key SARS-CoV-2 targets with molecular docking analysis.

A previous study explored the in vitro activities of over 69 flavonoids and their efficacy against SARS-CoV-2 using many SARS-CoV-2 targets, such as the SARS-CoV-2 3C-like protease (3CL^pro^), nucleocapsid (N) protein, the spike (S) protein–ACE2 interaction, papain-like protease (PL^pro^), and helicase [15]. The results detailed SARS-CoV-2 3C-like protease (3CL^pro^) as the most promising target, explaining the large number of flavonoids’ inhibitory activities against this protease. A recent study was performed on various flavonoids for screening their effect against coronavirus infection including quercetin, which showed the capacity to block the entry of SARS-CoV-2 in host cells [16]. Naringin aglycone (Naringenin) previously proved its activity against the hepatitis C virus (HCV) infection [17] and the alphavirus [18], while in our studies, we confirmed a direct role of naringin in the inhibition of the replication of the virus in lung cells pre-infection and even post-infection more than quercetin and other isolated phenolics. In SARS-CoV-2, molecular docking assumed that naringin has the power to inhibit SARS-CoV-2’s 3CL-M^pro^ and, thus, prohibit viral replication compared to the standard remdesivir, which is reported as a promising antiviral drug, similar to the ritonavir drug [19]. Many reports of compounds of natural sources with anti-SARS-CoV-2 potential are presently being investigated. Naringin flavonoid is the diglucoside of naringenin aglycone, which had been studied previously and proved to have potential effects against SARS-CoV-2.

Needless to say, all the prospective isolated compounds require in vivo anti-COVID-19 assessment and pharmacokinetic evaluation before going for clinical trial to find useful plant products for COVID-19 therapy, as elaborated in this article. Additionally, a species of *Garcinia* genus, *Garcinia kola*, was recently recorded for the treatment of respiratory disorders.

## 4. Conclusions

As shown in our study on *Garcinia cambogia* Roxb. fruit rind, it is rich in secondary metabolites, mainly phenolic compounds. Eight of them were isolated and subjected to in vitro anti-COVID-19 and in silico studies. Naringin was the most promising compound, followed by catechin and gallic acid; these results were further confirmed by the in silico experiment.

## 5. Materials and Methods

### 5.1. Plant Material

*Garcinia cambogia* Roxb. fruit rinds were purchased from a folk medicine market in Haram, Egypt in February 2022, confirmed by Asmaa Khamis, lecturer of Plant Taxonomy and the Flora-Department of Botany-Faculty of Science-Fayoum University. Specimen no. FuPD-2 was deposited at the department of Pharmacognosy, Faculty of Pharmacy, Fayoum University.

### 5.2. Preparation of Extracts

The dried *Garcinia cambogia* fruit rind (500 g) was well cleaned and milled by Moulinex caba (type 843, code 243, 220 vac 50 Hz 750 W) at 3000 RCF for 6 min at room temperature to obtain a homogenate powder, followed by extraction by cold maceration with 70% ethanol. The solvent was evaporated under reduced pressure at 40 °C to yield 42 g of dried crude total 70% EtOH extract, which was further used for metabolomic, phytochemical, and biological studies. The total EtOH extract of *Garcinia cambogia* Roxb. (40 g) was suspended in 250 mL water and successively fractionated using a separating funnel and solvents of increasing polarity: *n*-hexane (3.5 g), dichloromethane (1.1 g), ethyl acetate (1.2 g), *n*-butanol (2.2 g), and water (4.25 g). The remaining crude extract (2 g) was used for both metabolomic and biological studies.

### 5.3. Metabolomic Analysis

The total 70% EtOH extract of *Garcinia cambogia* Roxb. fruit rind under investigation was subjected to metabolomic analysis using LC-HRESIMS with the conditions previously described in the supplementary file, page S2 [20].

### 5.4. Phytochemical Study

A detailed chemical investigation from the plant materials to various chromatographic techniques has been applied to obtain the phenolic compounds to correlate phenolic compounds and biological activity. Details are mentioned in the supplementary file, page S3.

#### 5.4.1. Instruments and Reagents

For isolation, thin-layer chromatography (TLC), with precoated silica gel 60 F_254_ plates (Fluka−Sigma−Aldrich Chemicals, Schnelldorf, Germany), silica gel H for vacuum liquid chromatography (VLC), and silica gel 60 for column chromatography (CC) (E. Merck, Darmstadt, Germany), were used. Sephadex LH20 (Amersham Pharmacia Biotech AB, Uppsala, Sweden). For analysis, nuclear magnetic resonance, for ^1^HNMR, Bruker Avance III 400 MHz, and for ^13^CNMR, Bruker AG100 MHz with the BBFO Smart Probe (Switzerland), were all used.

#### 5.4.2. Solvent Systems

The following solvent systems were used for developing the TLC chromatography chromatograms, based on several trials: S_1_: CH_2_Cl_2_/MeOH; 9:1; S_2_: CH_2_Cl_2_/MeOH; 7.5:2.5; S_3_: CH_2_Cl_2_/MeOH; 8:2; S_4_: CHCl_3_/MeOH/H_2_O; 15:6:2; and S_5_: ethyl acetate/formic acid/acetic acid/H_2_O; 26:2.3:2.3:15.

### 5.5. Biological Study

#### 5.5.1. 3CL Protease Assay (M^Pro^ Assay)

According to the protocol of the manufacturer, the assay performed used SARS-CoV-2 proteases assay kits (M^Pro^, Catalog: 79955-1, BPS Bioscience, Inc., Allentown, PA, USA), using GC376 (MW = 507.5 Dalton) as the positive control. The assay principle is when the enzyme cleaves the substrate and leads to fluorescence production, measured using a microplate reader at wavelengths 460 for emission and 360 nm for excitation (Tecan Spark, Männedorf, Switzerland). In a 96-well plate, different concentrations of selected test compounds (10 μL) were mixed with 30 μL of diluted protease (15 μg/mL) were added, followed by the incubation of these mixtures at room temperature for half an hour. Afterward, a mixture of 10 μL of the substrate and the reaction buffer was added to the wells to attain a final volume of 50 μL with a final concentration of 40 μM. This final mixture was subjected to incubation at 20 °C for four hours using a fluorimeter (TECAN spark microplate-reading) in order to measure the produced fluorescence.

#### 5.5.2. Coronavirus COVID-19 (Genesig^®^ Real-Time PCR Assay)

Genesig^®^ Real-Time PCR assay is an in vitro diagnostic test developed to detect SARSal-Tim viral RNA. A sample was taken from nasopharyngeal and oropharyngeal swabs and sputum, processed under the World Health Organization (WHO) recommendations and according to the manufacturer’s instructions (Coronavirus COVID-19 Genesig^®^ Real-Time PCR Assay Issue 5.0, 2020, Primerdesign Ltd., Hampshire, United Kingdom). The extraction of samples with a volume of 700 µL each was performed using a CE IVD extraction system for the isolation of RNA with the aid of GXT DNA ⁄RNA extraction kits, followed by mixing the resuspended components with the reagents of the PCR reaction platform in an appropriate 96-well plate to perform the experiment. PCR assay results were interpreted and evaluated using the Applied Biosystem^®^ 7500 Real-Time PCR system.

- Kit Components:
  - Genesig^®^ easy RNA internal buffer extraction control
  - COVID-19 primer & probe Mix
  - Oasig™ resuspension buffer
  - Template preparation buffer
  - RNase/DNase-free water
  - Oasig™ lyophilized OneStep 2X RT-Qpcr Master Mix
  - COVID-19 positive control template
  - Appropriate nucleic extraction system and/or kit

- Equipment:
  - Real-Time PCR including Roche^®^ LightCycler 480 II from life science research, California, United States (software version 1.5), Applied Biosystem^®^ 7500 Real-Time PCR System (software version 2.3) and Detection System of Bio-Rad CFX Connect™ Real-Time PCR (Maestro software version 1.1) with Real-Time PCR System
  - White Bio-Rad CFX96 and White Roche^®^ Light Cycler 480.
  - Multiwell plate 96, adjustable pipettes, pipette tips with filters, transparent applied biosystems^®^ 7500, PCR hood, benchtop centrifuge, 1.5 mL microcentrifuge tubes, vortex mixer, and disposable gloves.

### 5.6. Molecular Docking Studies

Crystallographic structures of the COVID-19 protease enzyme in combination with its ligand are available in the Protein DataBank (PDB file 5R7Z) [21]. The revision of protein errors and protein structure creation were performed by the structure arrangement process on default rules on the molecular operating environment (MOE). Finally, protein partial charges were calculated by the Gasteiger methodology. Molecular docking analyses were performed with MOE2022 software [22]. The ligand coordinates were designed using the ChemDraw Ultra 11.0 program. Then, their protonation, the correction of the atom, and bond types were defined, followed by the hydrogen atoms addition, and finally minimization performance (MMFF94x, gradient: 0.01) [23]. The COVID-19 protease enzyme docking experiment was also accomplished by redocking the ligand in the PDB file 5R7Z, which was then deleted.

The default Triangle Matcher placement method for docking was processed by choosing a GBVI/WSA dG scoring function to determine the binding free energy of the ligand from a given pose to rank the final poses. The ligand complex with the enzyme possessing the lowest score was the best selection. The ligand redocking with its target discovered an RMSD 0.840 A°, which assured that the ligand bound to a similar pocket and confirmed the reliability of docking parameters.

### 5.7. Statistical Analysis

Biochemical data results have been recorded as means ± SE and then submitted to one-way ANOVA, comparing the means between groups using both the least-significant difference (LSD) and Duncan’s multiple range tests. The outcome results could be considered as statistically significant when *p*-value < 0.05 (Grapg Pad Prism 5).

## Figures and Tables

**Table 1:** Effect of isolated compounds on 3CL protease SARS-CoV-2 activity (M^Pro^ assay) compared to the plumbagin standard.

Compound	M^pro^	SD ±
Code	SARS-CoV-2 3CL Protease IC_50_ µg/mL	
1	42.26	2.3
2	73.3	3.98
3	99.75	5.42
4	34.43	1.87
5	16.62	0.9
6	26.2	1.42
7	77.26	4.2
8	30.35	1.65
Plumbagin	16.48	0.9

**Table 2:** Effect of compound 5 on COVID-19 compared to Remdesivir.

Sample Code	EC_50_ (µM)
Compound 5	0.0169
Remdesivir	0.0081

**Table 3:** The binding energy score of target compounds—COVID-19 protease enzyme complex binding conformations.

Compound No	Compound Name	Binding Energy Score	Average Number of Poses per Run *
1	Quercetin	−5.542	14
2	Amentoflavone	−6.701	16
3	Vitexin	−6.171	18
4	Rutin	No binding	
5	Naringin	−6.729	18
6	Catechin	−5.519	16
7	*P*-coumaric acid	−4.553	16
8	Gallic acid	−4.410	15

* The final score is the mean of three successive runs. * The docking method was confirmed by a successful pose-retrieval docking analysis of the ligand (score: −5.346).

## Data Availability

All datasets generated in this study are included in the article/supplementary material.

## Supplementary Materials

The following supporting information can be downloaded at https://www.mdpi.com/article/10.3390/plants11192521/s1. Figure S1: Photograph of *Garcinia cambogia* Roxb. Fruit; Figure S2: HPLC fingerprint of total 70% ethanol extract of *Garcinia cambogia* Roxb. fruits rind; Figure S3: Effect of isolated compounds on 3CL Protease SARS-CoV-2 Activity (M^Pro^ assay) compared to Plumbagin standard; Figure S4: Standard curves 3CL Protease SARS-CoV-2 Activity (MPro assay) on isolated compounds; Figure S5: Effect of compound 5 on Human coronavirus (COVID-19) compared to Remdesivir; Figure S6: Standard curves of effect of compound 5 on Human Coronavirus (COVID-19) compared to remdesivir; Table S1: Chemical profiling of the secondary metabolites of total 70% ethanol extract of *Garcinia cambogia* Roxb. fruits rind using LC-HR-ESI-MS; Table S2: Detailed 3CL Protease SARS-CoV-2 Activity (M^Pro^ assay) on isolated compounds; Table S3: Effect of *Garcinia cambogia* Roxb. isolated compounds on Human Coronavirus (COVID-19); Table S4: Evaluation of EC_50_ and percentage of inhibition of compound 5 on Human Coronavirus (COVID-19) compared to Remdesivir.
