# Supplementary material for: Garcinia cambogia Phenolics as Potent Anti-COVID-19 Agents: Phytochemical Profiling, Biological Activities, and Molecular Docking
Source: Plants (Basel). 2022 Sep 26;11(19):2521. doi: 10.3390/plants11192521 (PMC9572397; doi:10.3390/plants11192521)
Supplement: Supplementary file 1 [file plants-11-02521-s001.zip › plants-1916867-supplementary.pdf]

Supplementary

Garcinia cambogia phenolics as potent anti-COVID-19 agents: phytochemical profiling, biological activities and molecular docking

Hanan Y. Aati [1†], Ahmed Ismail [2†], Mostafa E. Rateb [3*], Asmaa M. AboulMagd [4], Hossam M. Hassan [5*], Mona H. Hetta [2]

[1] Department of Pharmacognosy, College of Pharmacy, King Saud University, P.O. Box 2457, Riyadh 11451, Saudi Arabia; e-mail: hati@ksu.edu.sa
[2] Pharmacognosy Department, Faculty of Pharmacy, Fayoum University, Fayoum, Egypt; e-mail: ais03@fayoum.edu.eg
[3] School of Computing, Engineering & Physical Sciences, University of the West of Scotland, Paisley PA1 2BE, Scotland, UK; e-mail: Mostafa.Rateb@uws.ac.uk
[4] Department of Pharmaceutical Chemistry, Faculty of Pharmacy, Nahda University (NUB), Beni-Suef, Egypt; asmaa.aboulmaged@nub.edu.eg
[5] Pharmacognosy Department, Faculty of Pharmacy, Beni-Suef University, Banī Suwayf, Egypt; e-mail: hossam.mokhtar@nub.edu.eg
[5] Pharmacognosy Department, Faculty of Pharmacy, Fayoum University, Fayoum, Egypt; e-mail: mhm07@fayoum.edu.eg
* Correspondence: Mostafa.Rateb@uws.ac.uk and hossam.mokhtar@nub.edu.eg
[†]**Equal contribution as first author**

Figure S1: Photograph of *Garcinia cambogia* Roxb. fruit**.**

Figure S2: HPLC fingerprint of total 70% ethanol extract of *Garcinia cambogia* Roxb. fruits rind.

Figure S3: Effect of isolated compounds on 3CL Protease SARS-CoV-2 Activity (M^{Pro} assay) compared to Plumbagin standard.

Figure S4: Standard curves 3CL Protease SARS-CoV-2 Activity (MPro assay) on isolated compounds.

Figure S5: Effect of compound 5 on Human coronavirus (COVID-19) compared to Remdesivir.

Figure S6: Standard curves of effect of compound 5 on Human Coronavirus (COVID-19) compared to remdesivir.

Table S1: Chemical profiling of the secondary metabolites of total 70% ethanol extract of *Garcinia cambogia* Roxb. fruits rind using LC–HR–ESI–MS.

Table S2: Detailed 3CL Protease SARS-CoV-2 Activity (M^{Pro} assay) on isolated compounds.

Table S3: Effect of *Garcinia cambogia* Roxb. isolated compounds on Human Coronavirus (COVID-19)**.**

Table S4: Evaluation of EC_{50} and percentage of inhibition of compound 5 on Human Coronavirus (COVID-19) compared to Remdesivir.

Metabolomic analysis

The total extract (1 mg/ml in MeOH) was uploaded on an Accela HPLC (Thermo Fisher Scientific, Bremen, Germany) combined with Accela UV/VIS and Exactive (Orbitrap) mass spectrometer from Thermo Fisher Scientific (Bremen, Germany). The mobile phase composed of water (A) and acetonitrile (B) with 0.1% formic acid in each solvent. The gradient elution started at a flow rate of 300 μL/min with 10% B linearly increased to 100% B within 30 min and remained isocratic for the next 5 min before linearly decreasing back to 10% B for the following 1 minute. The mobile phase was then equilibrated for 9 min before the next injection. The mass range was set from m/z (mass-to-charge ratio) 100–2000 for ESI–MS using in-source CID (collision-induced dissociation) mechanism and m/z 50–1000 for MS/MS using untargeted HCD (high energy collision dissociation). In MZmine 2.12, a framework for differential analysis of mass spectrometry data, the raw data were imported. Chromatogram deconvolution was then performed, followed by peaks deisotoping. For chromatographic alignment and gap-filling, the retention time normalizer was applied. Excel macros were used to combine positive and negative ionization mode data files generated by MZmine. Peaks produced from the sample were extracted. Excel macro was used to dereplicate each m/z ion peak with compounds in the customized database (using RT and m/z threshold of ±5 ppm), which provided details on the putative identities of all metabolites in the total extract. The excel macro was then utilized to identify the top 20 features (ranked by peak intensity) and the corresponding putative identities by creating a list for the extract. The

metabolites were then identified by comparison with some online and in-house databases.

Phytochemical study

An aliquot of the DCM fraction (1.1 g) was subjected to silica gel VLC (45 x 3cm, 50g) and eluted with *n*-hexane/Ethyl acetate mixture in 5% increment. Similar fractions were combined to give two subfractions (A1-A2). Subfraction A1 (150 mg, *n*-hexane/Ethyl acetate (50:50)) was rechromatographed on silica gel (60 cm × 1.4cm, 15g) with gradient elution using DCM/MeOH in 5 % increment using $\mathbf{S_1}$. The subfraction eluted with 20% MeOH in DCM was rechromatographed on Sephadex LH-20 and eluted with (DCM/MeOH; 1:1) to yield compound **1** (12 mg). The subfraction A2, (150 mg, *n*-hexane/Ethyl acetate (25:75)) was repurified using silica gel column (60cm × 1.4cm, 15g) and eluted with DCM/MeOH (1 % increment) using $\mathbf{S_2}$ and followed by Sephadex LH-20 (applying the band dissolved in DCM with least amount of MeOH) using 5% MeOH/DCM for elution, and compound **2** (10 mg) was obtained.

EtOAc fraction (1.2 g) was chromatographed on a silica gel column (52 cm x 3.5 cm, 35 g) using DCM/MeOH as an eluent in a gradient elution to yield two subfractions. The collected DCM/MeOH (95:5) eluate was subjected to another silica gel CC (30 cm x 1.2 cm, 10g) using $\mathbf{S_3}$ to give compound **2** (45 mg). The collected subfraction DCM/MeOH (65:35) eluate was further subjected to silica gel CC (45 cm x 2.5 cm, 40g) and eluted with the gradient solvent DCM/MeOH and using $\mathbf{S_4}$ which was further purified using

sephadex LH-20 (5cm x 1 cm, 6 g) and eluted with MeOH and resulted by compound **3** (10 mg). The *n*-BuOH extract (2.2 g) was suspended in 5 ml distilled water and poured on a polyamide column and eluted with H_2O (100%) and a mixture of H_2O/MeOH in a gradient elution (75, 50 and 25%). The fraction (H_2O/MeOH, 25%) was further rechromatographed on Diaion® HP-20 resin column (85cm x 3cm x 50gm) and eluted with H_2O/MeOH mixture ratio and the similar eluted subfractions were compiled together using **S_4** to obtain compound **4**,(15 mg) and compound **5**,(10 mg).

An aliquot of water fraction left after *n*-butanol (1.25 g) was partitioned on a CC using 50 g spherical regular silica (Sorbtech Norcross GA30071, USA) and isocratically eluted with 65 % DCM/MeOH using **S_4** and **S_5**, followed by RP-HPLC, to yield compound **6** (tR = 7.1 min, 15 mg).

The total phenolics content of *Garcinia cambogia* Roxb. fruit rind was extracted from the water fraction left after *n*-butanol fraction (3 g) using a previous reported method [25]. Two compounds were isolated; compound **7** (12 mg), and compound **8** (17 mg).

The following solvent systems were used for developing the TLC Chromatography chromatograms, based on several trials; S_1: CH_2Cl_2/MeOH; 9:1; S_2: CH_2Cl_2/MeOH; 7.5:2.5; S_3: CH_2Cl_2/MeOH; 8:2; S_4: $CHCl_3$/MeOH/H_2O; 15:6:2 and S_5: Ethyl acetate/formic acid/acetic acid/H2O; 26:2.3:2.3:15.

Table S1: Chemical profiling of the secondary metabolites of total 70% ethanol extract of *Garcinia cambogia* Roxb. fruits rind using LC–HR–ESI–MS.

Identified Metabolites	m/z	Retention time	Peak area (mzXML)	Conc. (µg/g)
Protocatechuic acid	155.11	7.02	1388.90	197.85
P- coumaric acid	164.06	10.88	3332.82	306.33
Vanillic acid	169.09	6.14	1505.50	245.20
Gallic acid	170.12	12.96	2569.58	198.27
Hydroxycitric acid	208.19	19.76	2437.11	123.34
Vomifoliol	225.15	22.84	6705.40	293.58
Naringenin	273.07	2.67	7857.69	2942.96
Isopimara-7,15-diene	273.26	27.84	2361.10	84.81
Isopimara-7,15-dien-19-ol	289.24	18.77	39355.33	2096.71
Catechin	290.79	17.6	525.73	29.87
Epicatechin	290.79	17.6	525.73	29.87
Quercetin	302.28	35.63	614.25	17.24
9,12-Dihydroxy-15-nonadecenoic acid	329.26	24.5	2272.10	92.74
Lariciresinol	361.17	18.74	1873.89	99.99
Glanduloidin C	399.18	8.24	572.04	69.42
Stigmasterol	413.36	19.41	536.46	27.64
β-sitosterol	415.36	20.32	816.49	40.18
Garcinoic acid	426.6	32.53	1917.79	58.95
Vitexin	432.38	34.08	2197.16	64.77
Prunin	434.41	19.45	413.29	21.25
Salvileucolide methyl ester	449.29	12.95	2079.73	160.60
Epigallocatechin-3-gallate	458.37	34.14	1155.74	33.85
Guttiferone Q	503.18	2.19	3023.71	1380.69
Guttiferone R	519.32	8.91	1713.46	192.31
Guttiferone S	519.32	8.91	1713.46	192.31
Hinokiflavone	539.09	35.54	2278.63	64.11
Amentoflavone	539.1	35.54	2278.63	64.11
Dihydrohinokiflavone	541.11	28.84	1363.47	47.28
Dihydroamentoflavone	541.12	28.84	1363.47	47.28
2,3-dihydrobilobetin	555.13	13.31	380.94	28.62
Isoginkgetin	567.13	12.13	438.49	36.15
β-Sitosterol-D-glucoside	577.46	15.89	5529.46	347.98
Naringin	581.15	35.46	1933.11	54.52
kaempferol 3-O-rutinoside	594.95	29.38	4353.49	148.18
Rutin	610.51	39.1	2731.20	69.85

* mzXML: Mass spectrometry data format

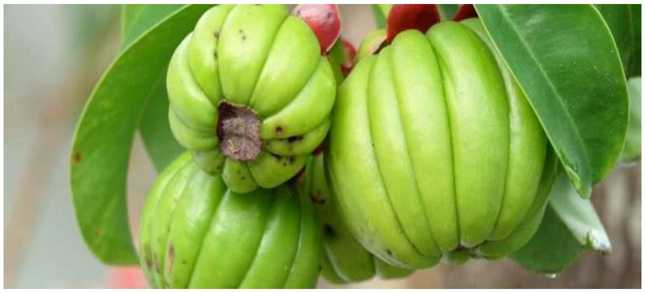

Figure S1: Photograph of *Garcinia cambogia* Roxb. fruit

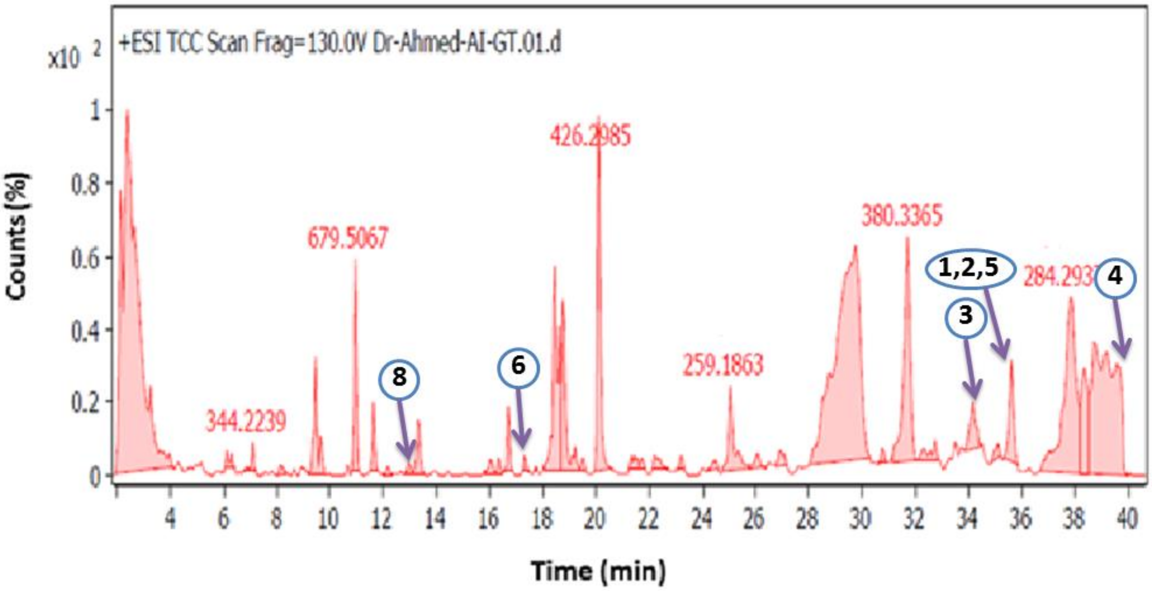

A

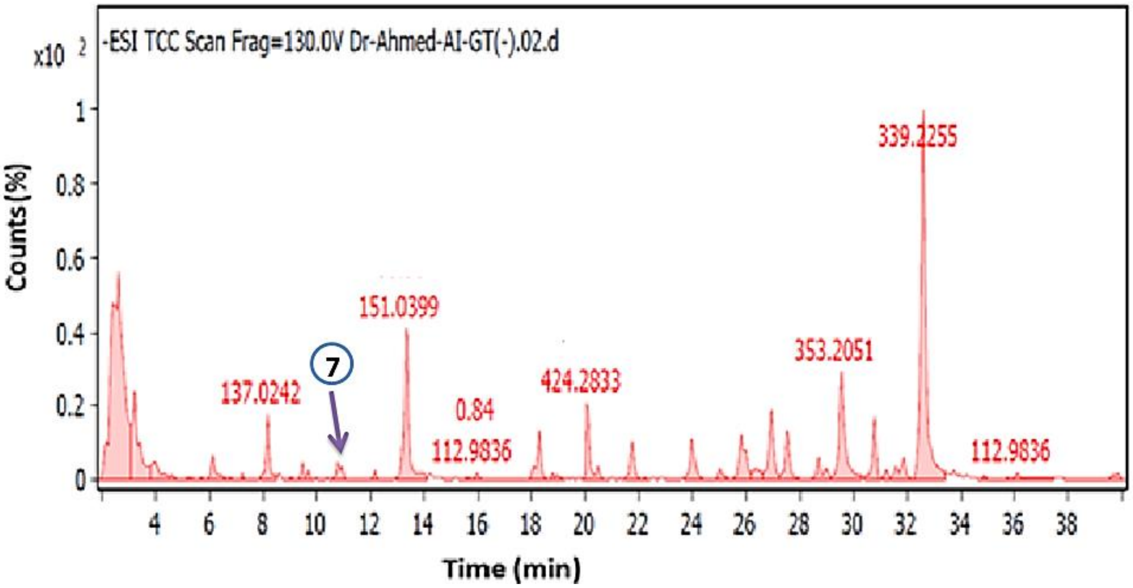

B

Figure S2: HPLC fingerprint of total 70% ethanol extract of *Garcinia cambogia* Roxb. fruit rind.
A: positive mode, B: Negative mode.

Table S2: Detailed 3CL Protease SARS-CoV-2 Activity (M^{Pro} assay) on isolated compounds (1-8) from *Garcinia cambogia* Roxb. Fruit and plumbagin positive control.

Code	Conc.	Log	%inh	T2	T1	ΔT	RFU2	RFU1	ΔRFU	Slope	Kinetic Activity
**1**	100	2	70.43	30	0	30	29.57	0	29.57	3.3333	35.4844
	10	1	21.54	30	0	30	78.46	0	78.46	3.3333	94.1529
	1	0	7.849	30	0	30	92.15	0	92.15	3.3333	110.581
	0.1	-1	3.239	30	0	30	96.76	0	96.76	3.3333	116.113
**2**	100	2	62.98	30	0	30	37.02	0	37.02	3.3333	44.4244
	10	1	21.04	30	0	30	78.96	0	78.96	3.3333	94.7529
	1	0	5.849	30	0	30	94.15	0	94.15	3.3333	112.981
	0.1	-1	2.079	30	0	30	97.92	0	97.92	3.3333	117.505
**3**	100	2	61.05	30	0	30	38.95	0	38.95	3.3333	46.7405
	10	1	17.39	30	0	30	82.61	0	82.61	3.3333	99.133
	1	0	8.119	30	0	30	91.88	0	91.88	3.3333	110.257
	0.1	-1	2.409	30	0	30	97.59	0	97.59	3.3333	117.109
**4**	100	2	73.08	30	0	30	26.92	0	26.92	3.3333	32.3043
	10	1	22.58	30	0	30	77.42	0	77.42	3.3333	92.9049
	1	0	8.279	30	0	30	91.72	0	91.72	3.3333	110.065
	0.1	-1	3.269	30	0	30	96.73	0	96.73	3.3333	116.077
**5**	100	2	78.48	30	0	30	21.52	0	21.52	3.3333	25.8243
	10	1	33.14	30	0	30	66.86	0	66.86	3.3333	80.2328
	1	0	15.03	30	0	30	84.97	0	84.97	3.3333	101.965
	0.1	-1	1.969	30	0	30	98.03	0	98.03	3.3333	117.637
**6**	100	2	72.25	30	0	30	27.75	0	27.75	3.3333	33.3003
	10	1	30.64	30	0	30	69.36	0	69.36	3.3333	83.2328
	1	0	10.69	30	0	30	89.31	0	89.31	3.3333	107.173
	0.1	-1	5.039	30	0	30	94.96	0	94.96	3.3333	113.953
**7**	100	2	64.94	30	0	30	35.06	0	35.06	3.3333	42.0724
	10	1	16.39	30	0	30	83.61	0	83.61	3.3333	100.333
	1	0	5.079	30	0	30	94.92	0	94.92	3.3333	113.905
	0.1	-1	1.269	30	0	30	98.73	0	98.73	3.3333	118.477
**8**	100	2	75.14	30	0	30	24.86	0	24.86	3.3333	29.8323
	10	1	22.42	30	0	30	77.58	0	77.58	3.3333	93.0969
	1	0	8.969	30	0	30	91.03	0	91.03	3.3333	109.237
	0.1	-1	2.079	30	0	30	97.92	0	97.92	3.3333	117.505
**Plumbagin**	100	2	77.48	30	0	30	22.52	0	22.52	3.3333	27.0243
	10	1	35.04	30	0	30	64.96	0	64.96	3.3333	77.9528
	1	0	14.29	30	0	30	85.71	0	85.71	3.3333	102.853
	0.1	-1	4.109	30	0	30	95.89	0	95.89	3.3333	115.069
*** Post cont.**			0	30	0	30	100	0	100	3.3333	120

* Post. cont.: COVID-19 Positive control template

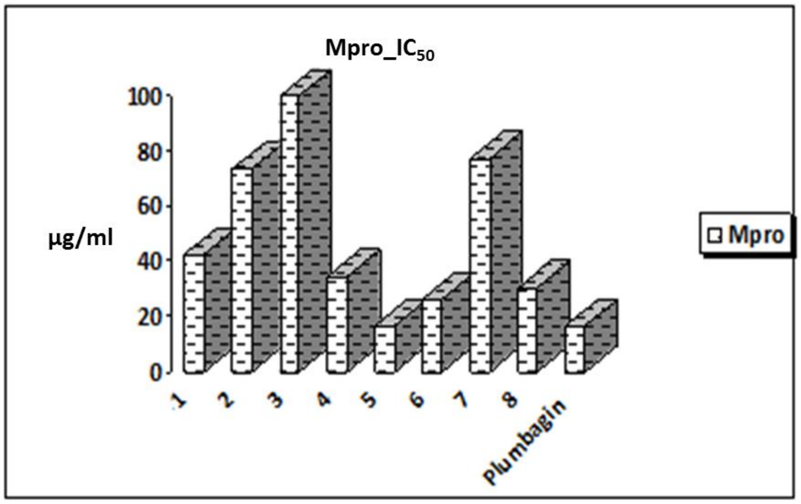

Figure S3. Effect of isolated compounds on 3CL Protease SARS-CoV-2 Activity (M^{Pro} assay) compared to Plumbagin standard.

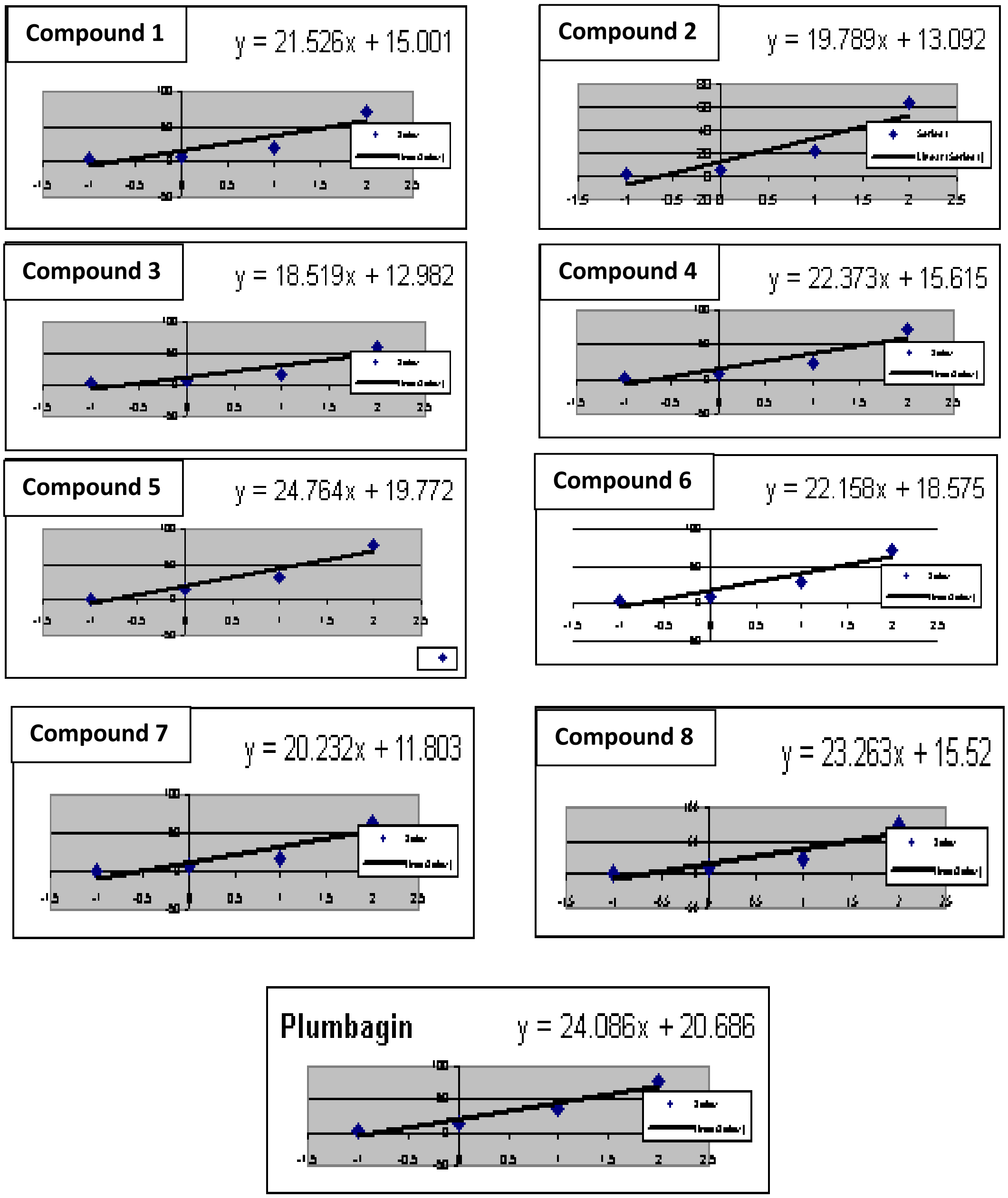

Figure S4: Standard curves 3CL Protease SARS-CoV-2 Activity (MPro assay) on isolated compounds (1-8) from *Garcinia cambogia* Roxb. Fruits.

X axis: Conc. (log/ μM) , Y axis: Time (ΔT)

Table S3: Effect of *Garcinia cambogia* Roxb. isolated compounds on Human Coronavirus (COVID-19)

Sample		Viral Titer			Inhibition	Reduction	Log Difference
Code	Conc. μM	CT	IU/ml	Log	%	%	
1	10	25.46	191047.1964	5.281140669	87.91045	14.80286757	-0.917589873
	1	23.88	572856.6142	5.758045932	63.749384	7.109271923	-0.44068461
2	10	25.16	235338.1316	5.371692301	85.107701	13.34205827	-0.827038241
	1	23.43	783201.2648	5.89387338	50.438683	4.918057979	-0.304857162
3	10	23.61	691102.3995	5.839542401	56.266739	5.794543553	-0.359188141
	1	22.75	1256387.904	6.099123746	20.495227	1.606890239	-0.099606796
4	10	23.95	545653.8422	5.736917218	65.470787	7.450127423	-0.461813324
	1	22.81	1205073.177	6.08101342	23.742445	1.899052091	-0.117717122
5	10	27.86	36035.25597	4.556727611	97.719673	26.48934197	-1.642002931
	1	25.95	135906.3252	5.13323967	91.399789	17.18885609	-1.065490872
6	10	25.64	168581.413	5.22680969	89.332095	15.67935314	-0.971920852
	1	22.71	1291806	6.111197297	18.253954	1.412115665	-0.087533245
7	10	25.79	151891.6408	5.181533873	90.388231	16.40975781	-1.017196669
	1	24.26	439893.1485	5.643347198	72.16337	8.959630367	-0.555383344
8	10	26.06	125903.3844	5.100037405	92.032779	17.72448616	-1.098693137
	1	23.78	614086.8665	5.788229809	61.140316	6.622335496	-0.410500733
Remdesivir	10	28.66	20666.00795	4.315256592	98.692246	30.38483343	-1.88347395
	1	26.86	72204.85253	4.858566385	95.430846	21.61997764	-1.340164157
* Post. cont.		22.42	1580267.258	6.198730542	0	0	0

* Post. cont.: COVID-19 Positive control template
* IU/ml: International unit per millimeter

Table S4: Evaluation of EC_{50} and percentage of inhibition of compound 5 on Human Coronavirus (COVID-19) compared to Remdesivir.

Sample		Viral Titer			Inhibition	Reduction	Log diff	EC50
Code	Conc. μM	CT	IU/ml	Log	%	%		
Compound 5	10	27.86	36035.25597	4.556727	97.268035	25.54673	-1.56352	0.016997
	1	25.95	135906.3252	5.13323	89.696442	16.12699	-0.987012	
	0.1	24.75	312929.2783	5.49544	76.275682	10.20883	-0.624806	
	0.01	23.46	767040.3613	5.88481	41.847853	3.84680	-0.235434	
	0.001	23.03	1034210.374	6.01460	21.592713	1.72613	-0.105643	
Remdesivir	10	28.36	25457.05872	4.405808	98.070007	28.01263	-1.714444	0.008182
	1	27.11	60688.5403	4.783106	95.398979	21.84788	-1.337145	
	0.1	26.44	96680.45161	4.98533	92.6703	18.54357	-1.134913	
	0.01	23.84	589005.6799	5.77011	55.345316	5.72089	-0.350132	
	0.001	22.96	1085769.415	6.03573	17.683832	1.38090	-0.084514	
* Post. cont.	---	22.68	1319023.28	6.12025	0	0	0	

* Post. cont.: COVID-19 Positive control template
* IU/ml: International unit per millimeter

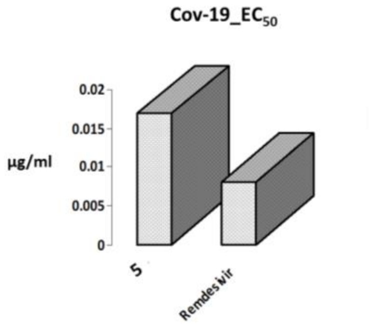

Figure S5. Effect of compound 5 on Human coronavirus (COVID-19) compared to Remdesivir.

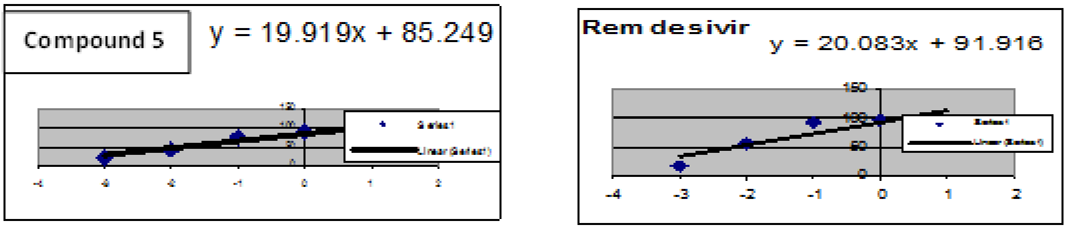

Figure S6: Standard curves of effect of compound 5 on Human Coronavirus (COVID-19) compared to Remdesivir.

X axis: Conc. (log/ μM) , Y axis: Time (ΔT)
